# Improvements toward highly accurate diffraction experiments at the macromolecular micro-crystallography beamline BL-17A

**DOI:** 10.1107/S0909049513022875

**Published:** 2013-10-08

**Authors:** Yusuke Yamada, Leonard M. G. Chavas, Noriyuki Igarashi, Masahiko Hiraki, Soichi Wakatsuki, Naohiro Matsugaki

**Affiliations:** a Photon Factory, Institute of Materials Structure Science, High Energy Accelerator Research Organization, 1-1 Oho, Tsukuba, Ibaraki 305-0801, Japan; b Photon Science, SLAC, 2575 Sand Hill Road, MS 69, Menlo Park, CA 94025-7015, USA; c Structural Biology, School of Medicine, Stanford University, 279 Campus Drive, Stanford, CA 94305-5126, USA

**Keywords:** macromolecular crystallography, beamline

## Abstract

BL-17A is a macromolecular crystallography beamline dedicated to diffraction experiments conducted using micro-crystals and structure determination studies using a lower energy X-ray beam. In these experiments, highly accurate diffraction intensity measurements are definitively important. Since this beamline was constructed, the beamline apparatus has been improved in several ways to enable the collection of accurate diffraction data. The stability of the beam intensities at the sample position was recently improved by modifying the monochromator. The diffractometer has also been improved. A new detector table was installed to prevent distortions in the diffractometer’s base during the repositioning of the diffractometer detector. A new pinhole system and an on-axis viewing system were installed to improve the X-ray beam profile at the sample position and the centering of tiny crystal samples.

## Abstract 2

The BL-17A macromolecular crystallography beamline at the Photon Factory was updated to improve the accuracy of diffraction experiments conducted using tiny crystals.

## 1. Introduction

Macromolecular micro-crystallography has evolved rapidly and become a standard tool for macromolecular crystallography (Evans *et al.*, 2011 ▶; Smith *et al.*, 2012 ▶). The Photon Factory includes five beamlines for macromolecular crystallography studies. Among the beamlines, BL-17A is the first beamline dedicated to micro-crystal diffraction studies and structure determinations using a lower-energy X-ray beam (Igarashi *et al.*, 2007 ▶). Several significant results have been reported since the beamline’s operations commenced in 2006. Certain biological targets can be difficult to analyze, and the beamline performance requirements have increased over time. The sizes of crystals that users bring to the beamline are becoming smaller, necessitating more stable beams. The beamline must be improved to respond to these requirements (Igarashi *et al.*, 2008 ▶). This article describes several recent improvements in the beamline instrumentation that enable highly accurate diffraction experiments at BL-17A.

## 2. Beamline configuration

The BL-17A light source is a newly developed short-gap undulator installed at the Photon Factory 2.5 GeV storage ring (Yamamoto *et al.*, 2007 ▶). The main optical components of BL-17A are the vertically deflecting double-crystal monochromator (DCM) and two mirrors that focus the X-ray beams vertically and horizontally. The DCM is positioned 17.5 m from the light source. The vertical and horizontal focusing mirrors are positioned 22.2 m and 29.5 m, respectively, from the light source with demagnification ratios of 1.6:1 and 4.2:1. A high-precision diffractometer equipped with an air-bearing goniometer head, a high-speed shutter and a CCD area detector (ADSC Quantum 270) is positioned so that the rotational axis of the sample was located 36.0 m from the light source. For beam diagnostics, two beam position monitors are installed. The UHV QBPM, manufactured by FMB Oxford, is positioned 21.5 m from the light source to accept a monochromatic beam from the DCM. The other position monitor is a RIGI manufactured by Dectris and positioned 35.5 m from the light source to accept a focus beam from the two mirrors.

This configuration permits the beamline to deliver a 250 µm × 50 µm (H × V) focused beam at the sample position. A typical experiment is carried out using a 50 µm × 50 µm beam defined by a four-blade slit placed 50 mm upstream from the sample position. The flux of the 50 µm × 50 µm beam was 5.5 × 10^10^ photons s^−1^ at 12.6 keV.

## 3. DCM improvements

The DCM is one of the most important components for stabilizing the intensities and positions of the monochromatic X-ray beams at the sample position. The DCM at BL-17A is equipped with a liquid-nitrogen cooling system to eliminate the large quantity of heat load dumped on the crystals. Inside the chamber are tubes to permit the liquid-nitrogen flow and in some parts flexible tubes were used. The flexible tube from the original DCM had a bellow shape, which may cause a turbulent liquid-nitrogen flow. Consequently, vibrations of the crystals occurred and this resulted in fluctuations in the beam intensity and position at the sample position. The turbulent flow was suppressed by replacing the tubes with new tubes (Clear Flow Flex) that were originally developed by RIKEN and JASRI in SPring-8 and commercialized by Osaka Rasenkan Kogyo. The new tube is equipped internally with a specially designed flexible sleeve, which suppresses a turbulent flow to reduce the vibration caused by a fluid running through the tube.

**Figure 1:**
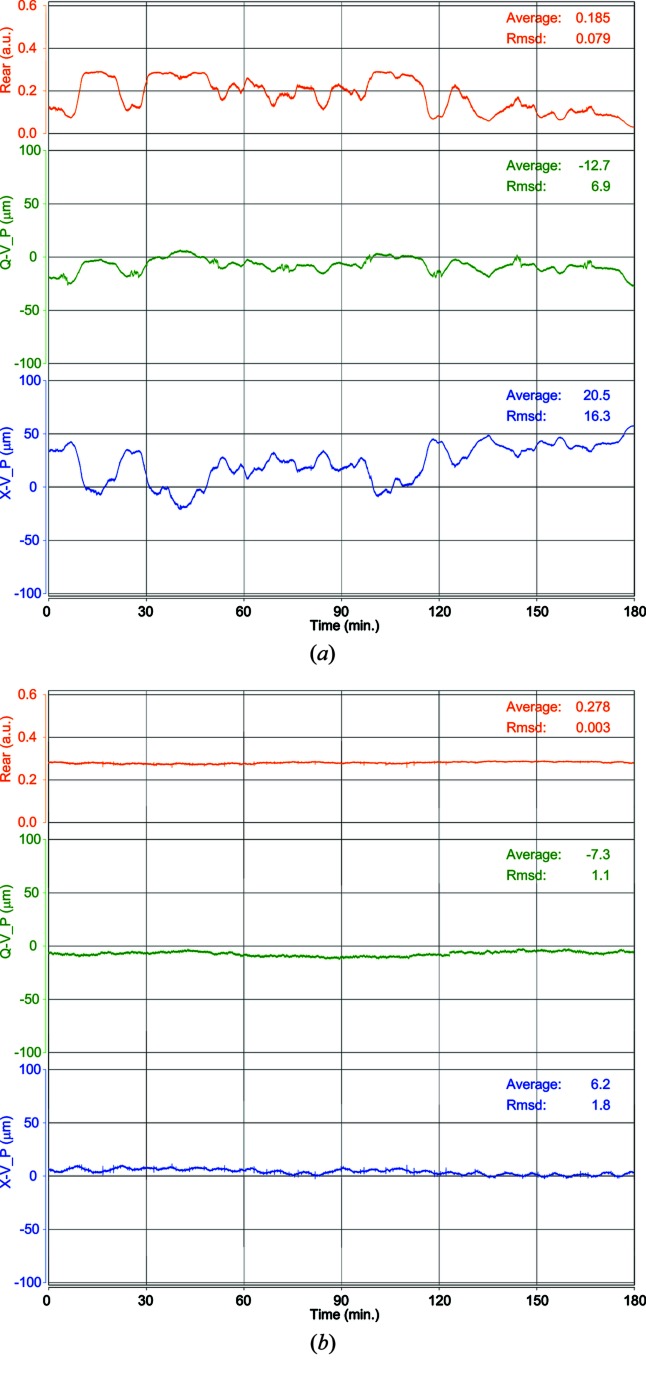
Stabilization of the X-ray beam exiting from the DCM. (*a*) Watlow controller, (*b*) Lakeshore controller. In each plot, Rear, Q-V_P and X-V_P represent the beam intensity through a pinhole 20 µm in diameter at the sample position, the vertical position of an X-ray beam measured by UHV QBPM, and the vertical position of an X-ray beam measured by RIGI, respectively. The average and the standard deviation of each plot were calculated with the first 30 min data.

The crystals in the DCM are cooled to liquid-nitrogen temperature; however, they are mounted on motorized stages that need to be kept sufficiently warm to move smoothly. The stages are kept warm by placing heaters and insulators between the crystal holders and the motorized stages. Since the beamline was built and its user operation commenced, we have utilized a Watlow Series 93 heater controller; however, this heater controller caused fluctuations in the X-ray beam intensity at the sample position. This controller intermittently delivers current to a heater and controls the temperature by changing the current level and frequency. Although we carefully tuned these parameters to suppress fluctuations in the beam intensity, there was still a long-term drift of the vertical position of the beam exiting from the DCM and the beam intensity at the sample position (Fig. 1*a* ▶). This drift corresponded to about a 50 µm shift in 10 min at the sample position. The temperature was more precisely stabilized by replacing the controller with a Lakeshore Model 335. This model can deliver current to the heater continuously and control the temperature by changing only the current level more finely compared with the previous controller. The beam drift was drastically suppressed once the controller had been replaced (Fig. 1*b* ▶). Prior to changing the heater controller, the beam position feedback system had been necessary for stabilizing the beam intensity at the sample position (Igarashi *et al.*, 2008 ▶). The new controller obviated the need for the beam position feedback system because the beam intensity at the sample position remained stable enough during data collection.

## 4. Diffractometer improvements

### 4.1. A new detector table

**Figure 2:**
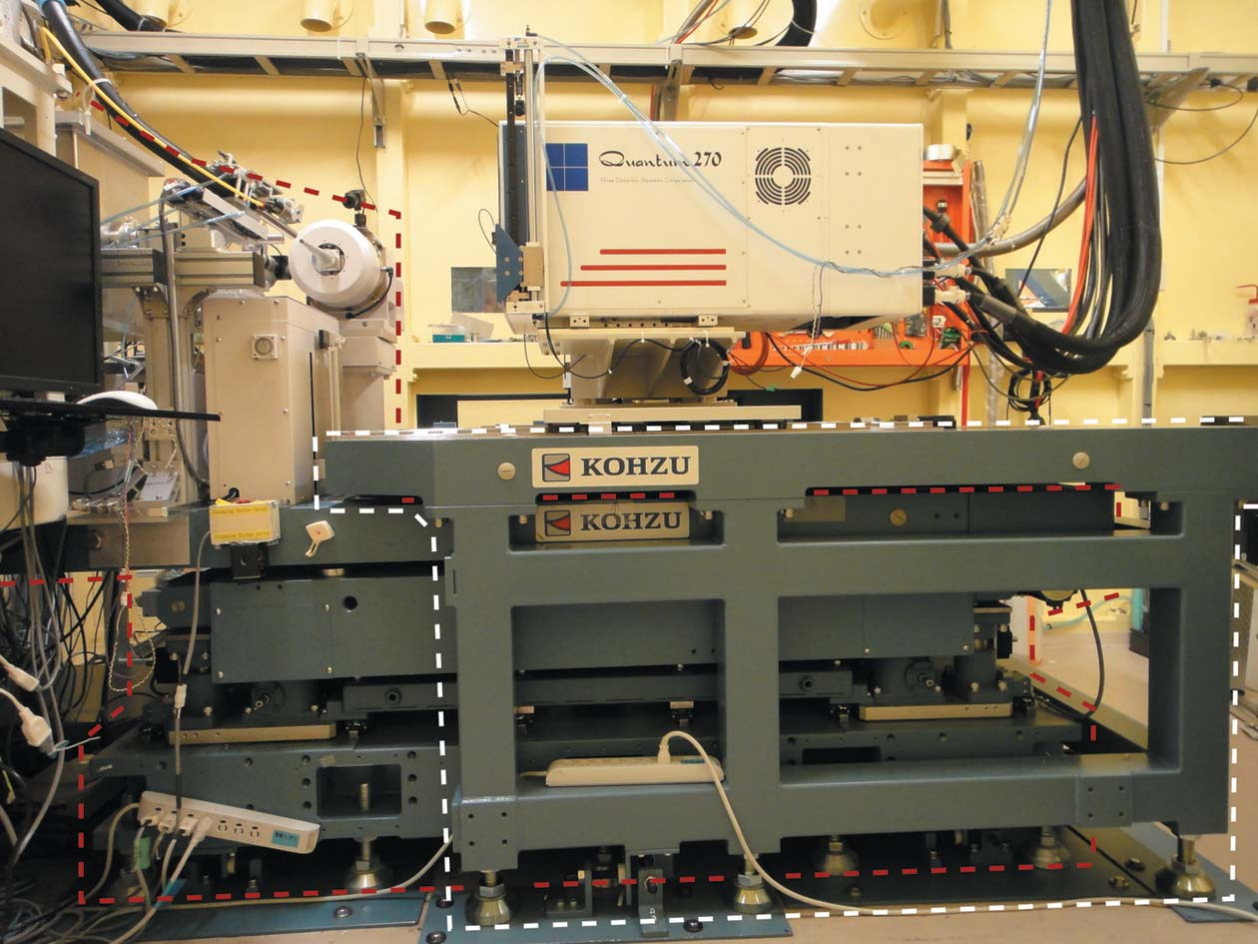
The new detector table and the existing diffractometer are highlighted by white and red dotted lines, respectively.

The diffractometer at BL-17A was originally designed based on those developed at other macromolecular crystallography beamlines, BL-5A and AR-NW12A, at the Photon Factory. Like these other diffractometers, the base plate of the diffractometer was also used to support a table for the CCD area detector as well as all of the components, including the beam-defining slits, the beam position monitor and the goniometer head. After construction had been completed, it was found that, as the detector was moved to change the distance between the sample and the surface of the detector, the base plate became distorted and the relative positions between the components and the X-ray beam had changed. The displacements due to the various components differed and reached a maximum of about 20 µm. At the BL-5A and AR-NW12A beamlines, the displacements were acceptable because the size of the focused beams was around 200 µm in the vertical direction; however, the displacements were not acceptable at BL-17A because these distances corresponded to the size of the focused beam. This problem was addressed by designing and installing a new detector table. The new detector table had a structure that allowed it to stride across the diffractometer (Fig. 2 ▶) while maintaining a complete separation from the diffractometer. The movements of the detector no longer influenced the displacements of components on the diffractometer.

### 4.2. A new collimator

**Figure 3:**
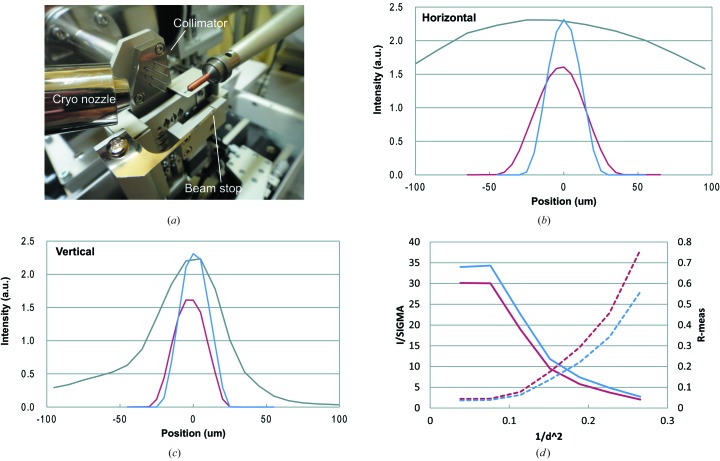
The new collimator. (*a*) Photograph of the new collimator installed on the diffractometer of BL-17A. (*b*), (*c*) Comparison of the horizontal and vertical beam profiles at the sample positions. Gray, red and blue represent the profiles of a full-sized focus beam, a beam defined by a slit and a beam defined by the collimator, respectively. The beam profiles were measured by scanning the beams by using a pinhole 20 µm in diameter at the sample position. Fluxes of the beams defined by the slit and the collimator are 2.2 × 10^9^ and 1.7 × 10^9^ photons s^−1^, respectively, at 12.6 keV. (*d*) Comparison of the data collection statistics (solid: *I*/σ; dash: *R*_meas_) of a thaumatin crystal. Red and blue indicate the statistics of the data sets collected using the slit or collimator, respectively. The data sets were processed using *XDS* (Kabsch, 2010 ▶).

BL-17A delivers a focused beam with a focal size of 250 µm × 50 µm at the sample position. On the diffractometer was a four-blade slit located 50 mm upstream from the sample position. This slit defined the actual size of the beam exposed to the sample. In most cases, the user used a 50 µm × 50 µm aperture; however, the difficulties associated with defining the beam size were less than 30 µm. We therefore installed a new collimator to define beams smaller than 30 µm (Fig. 3*a* ▶). The design of the new collimator was inspired by the minibeam collimator developed at the GM/CA-CAT beamlines at the APS (Fischetti *et al.*, 2009 ▶). A unit of the new collimator at the BL-17A consisted of a 2 mm-diameter tantalum disk with a pinhole in the center and a stainless steel tube, the inner diameter of which was 0.3 mm. This unit acted as a scatter guard. Six units were situated on a support block, and four of these units were aligned along a line inclined at an angle of 60° from a horizontal plane and the others were aligned along another line. This inclination prevented collisions between the collimator tubes and the tip of the cryo nozzle which is positioned 4 mm away from the sample position during data collection and inclined at an angle of 30° from the horizontal plane. Consequently, the collimator could be positioned 15–20 mm upstream from the sample position. The former four units had different pinhole sizes, 10 µm, 30 µm, 50 µm and 80 µm, and a 10 mm tube. These four units were used in the user’s experiment. The latter two units included 50 µm pinholes, one of which not having a tube and the other of which had a 5 mm-length tube. These units were used to initially align the support block. The support block was attached to a motorized four-axis stage, and its vertical and horizontal translations and two orientations, yaw and pitch, could be adjusted precisely. In the beamline control software, the user could select a pinhole size for the diffraction experiment.

Figs. 3(*b*) and 3(*c*) ▶ show profiles of a focused beam as well as beams defined by the old four-blade slit and the new collimator. The apertures of the slit and collimator were 30 µm. The beam defined by the slit displayed a lower-intensity peak than the focused beam. The larger full width at half-maximum (FWHM) of the profile compared with the aperture size. On the other hand, the beam defined by the collimator was almost the same intensity as the focused beam, and the FWHM was the same as the aperture size. The new collimator defined a beam size at a position closer to the sample than the older four-blade slits. This explained why the beam profile at the sample position was improved.

Fig. 3(*d*) ▶ shows a comparison of the data collection statistics for a thaumatin crystal sample, collected using a beam defined by the slit and the collimator. In these experiments the aperture of the slits and the collimator were 50 µm. Both data sets were collected from the same crystal. The effects of radiation damage were eliminated by collecting data sets using the old four-blade slits. A new collimator was then collected. Despite the fact that the data set collected using the collimator was collected later, the data collection statistics were better across the full range of resolutions. This result suggested that improvements in the beam profile improved the data collection statistics. It should be noted that the beam shapes defined by the slits and collimator were rectangular and circular, respectively. This may also have contributed to the improved data collection statistics.

### 4.3. On-axis viewing system

**Figure 4:**
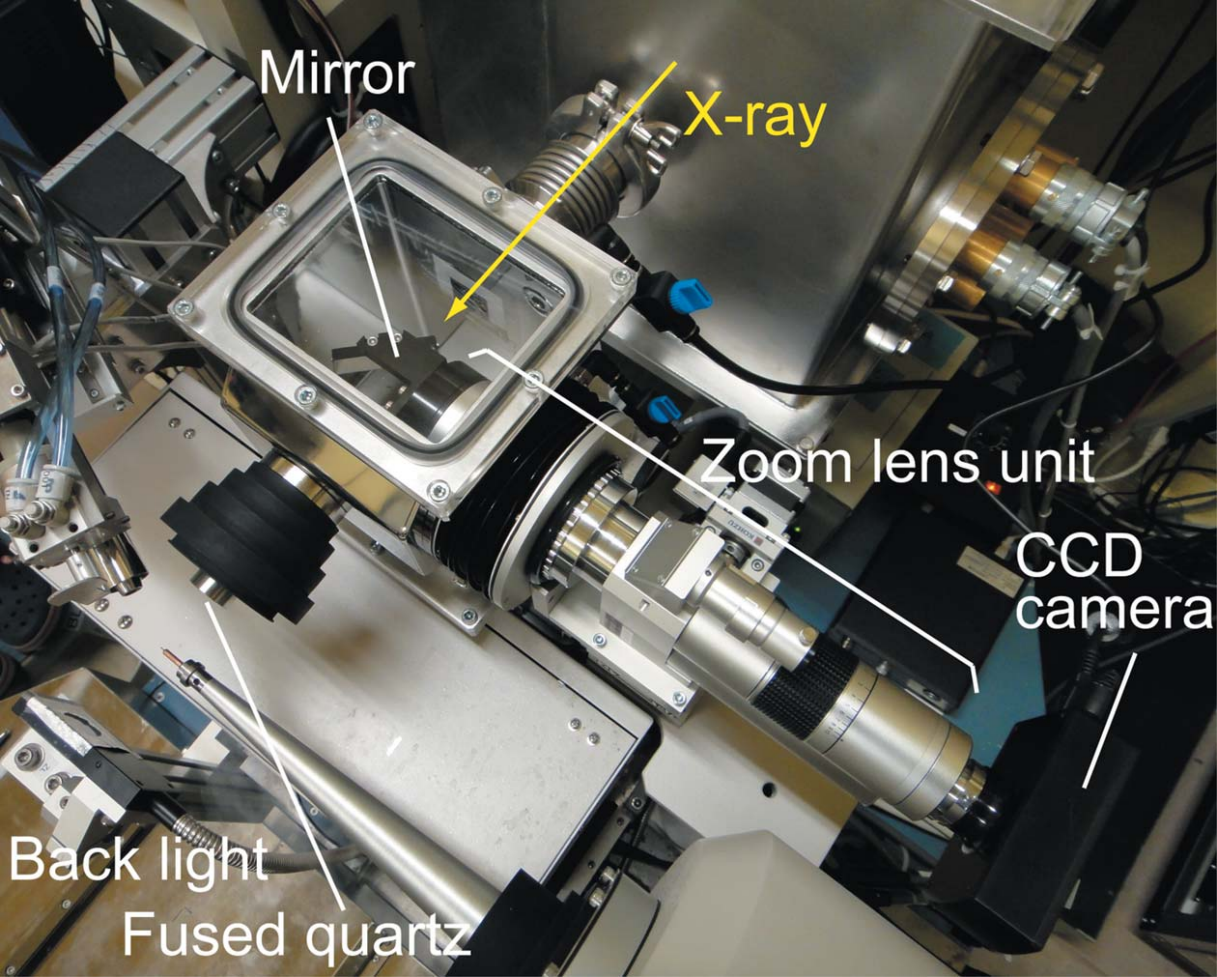
The new on-axis viewing system. It is noted that the new collimator unit was evacuated into a box under the spindle axis in this picture.

The diffractometer at BL-17A originally included a sample viewing system that was vertically tilted at a 60° angle relative to the X-ray beam direction. The spindle axis on a diffractometer is precise. All displacements during rotation of the spindle axis are less than 0.6 µm. The inconsistency between the directions of the X-ray beam and the sample viewing system were not expected to present a problem for the sample centering. This viewing system, however, had several dis­advantages. For example, the beam conditions could not be observed directly by illuminating a YAG scintillator placed at the sample position. Relations between the diffraction geometry and orientations and shape of a sample were difficult to understand. The first issue had important implications on the tuning of the X-ray beam at the sample position by visual inspection. Visual feedback is important for understanding the behaviors of the optical components and for aligning these components precisely to provide a stable beam at the sample position. These problems were addressed by installing a new on-axis viewing system. The new on-axis viewing system consisted of a zoom lens, an industrial CCD camera, a support with adjusting stages, and a chamber (Fig. 4 ▶). The zoom lens is the ULWZ-200M from Sigma Koki. This lens has a working distance of 205 mm and a maximum numerical aperture of 0.2. In front of an objective lens, a mirror is placed to reflect an image at 90°. A 1.5 mm-diameter hole is punched in the mirror to allow the X-ray beam to pass through. The long working distance enables the zoom lens unit to be placed about 150 mm away from the sample position to create a large space around the sample position. This space was important for installing a helium path around the sample that could be used to conduct diffraction experiments using a lower-energy X-ray beam. The zoom lens was fixed to the diffractometer by a support with a three-axis stage. The stage allowed the zoom lens to be adjusted in the *XYZ* directions. The zoom lens was inserted into the chamber, which was then filled with helium gas to maintain the beam intensity by reducing the absorption by air. The sample end of the chamber was sealed using an 8 µm-thick fused quartz plate, which was sufficiently transparent to use for both an X-ray beam and visible light.

Replacing the sample viewing system facilitated the alignment of the optical components. A user could understand the relationship between the diffraction patterns and the sample shape and orientation intuitively, which left them free to concentrate more on sample evaluations.

## 5. Summary

We have described the recent improvements of the beamline apparatus at BL-17A, the macromolecular micro-crystallography beamline at the Photon Factory. Replacing the flexible tubes and heater controller in the DCM yielded a much more stable monochromatic beam from the DCM. By installing a new apparatus on the diffractometer, higher quality beams could be obtained at a given position. The new collimator performed particularly well in defining the beam size (less than 30 µm) and obtaining a higher quality data set compared with the old four-blade slits.

These improvements led to the identification of other problems that had not been appreciated prior to the improvements. Fig. 1(*b*) ▶ shows the results of a small and periodical beam position displacement by the beam position monitor on the diffractometer. This displacement arose from thermal changes in the experimental hutch in which the diffractometer was placed. The installation of a new air-conditioning system permitted the temperature around the diffractometer to be controlled more precisely. This displacement may be reduced. Another characteristic feature of BL-17A is that it provides intense lower-energy X-ray beams for structural studies using anomalous signals from light atoms. In such experiments, highly accurate measurements are necessary because the measured signals are very weak. In order to make this a more routine phasing method, further improvements and developments are in progress.
